# In This Issue

**DOI:** 10.1002/1097-0061(20000930)17:3<167::AID-YEA31>3.0.CO;2-1

**Published:** 2000


**Yeast**
*Yeast* 2000; **17**: 167–168.

### In this Issue

### The dual origin of the yeast mitochondrial proteome

In their paper, Karlberg *et al.* describe a scheme for the origin of the mitochondrial protein complement, based on phylogenetic reconstructions involving over 400 yeast nuclear genes that encode mitochondrial proteins. They believe that a free-living $\alpha$-proteobacterium evolved into an endosymbiont of an anaerobic host, losing most of its ancestral bacterial genes, and eventually transferring some with roles in bioenergetic and translational processes to the nuclear genome. The endosymbiont also gained a large number of genes from the nuclear genome, which transformed it into an ATP-exporting organelle.

### Peroxisomal $\beta$-oxidation — *C. elegans* as the new model

Neurodegeneration is a common symptom of several human peroxisomal disorders, but the role of peroxisomal processes in the maintenance of neurons is not completely known. The nematode neural network is well characterized and relatively simple in function, thus *C. elegans* is a possible model for the study of this neurodegeneration. Gurvitz *et al.* have used homology searches to identify nematode orthologues of yeast $\beta$-oxidation enzymes and *C. elegans* PEX-5, the receptor for peroxisomal targeting signal type 1. PSORT II was used to predict the subcellular localizations of the genes, and the *C. elegans* PEX-5 was used in two-hybrid screens, to verify the predicted peroxisomal location of several of the nematode $\beta$-oxidation enzymes.

### Global cDNA amplification combined with real time RT–PCR

One factor currently limiting the breadth of application of microarray technology is the requirement for micrograms of starting, total RNA for the production of labelled targets. Samples yielding low levels of RNA, such as clinical samples of diseased or infected tissues, do not provide anything like these levels of RNA. Al-Taher *et al.* present a strategy for the amplification of small-scale samples, whilst maintaining the relative representation of each message within the sample. The approach has been adapted to allow use with TaqMan™ real time quantitative PCR, and in this study, applied to the quantitation of potassium channel mRNAs in a range of tissue types and in small numbers of cells grown in culture.

### Expression profiling at the single cell level — small is beautiful

In his review, Ged Brady discusses the methods currently available for the assessment of expression levels of multiple genes in small-scale samples down to single-cells. The review provides information on the technical aspects of each method, highlighting their strengths and weaknesses, and goes on to describe the fields of biological research that have benefited from the application of these methods.

### How many human genes are there? — the gene guessing game

Double Twist's recent human gene count prediction, based on the ~90% of the genome held in the public databases, followed by the joint announcement of the near-completion of the human genome has sparked a lively debate as to how many genes our genome encodes. Ian Dunham assesses each of the estimates made public so far, from those over 100 000 from Incyte and TIGR, to those around 35 000 arising from his own study of chromosome 22 and other strategies, such as comparison with the pufferfish, *Tetraodon nigroviridis*.

### Featured organism — the zebrafish, *Danio rerio*

In this issue we feature the zebrafish, a model organism close to the heart of many researchers working on development, particularly those with an interest in organ development. The zebrafish has

Copyright © 2000 John Wiley & Sons, Ltd.

made a huge contribution to our understanding of these areas, due in the main part to genetic screens of large panels of mutagenized fish, done in parallel by Christiane Nüsslein-Volhard, Wolfgang Driever and Mark Fishman's laboratories. On the eve of a second screen, aimed at achieving saturation of developmental genes, we discuss the progress made so far and take in comments on the future of zebrafish genomics research from Mark Fishman and Stephen Wilson.

### Interview — Stefan Schulte-Merker

Artemis Pharmaceuticals has recently launched a second mutagenesis screen of the zebrafish, with the aim of detecting genes with roles in development that were missed in the first screens. We discuss the results of the first screens and the plans for the second screen with Stefan Schulte-Merker of Artemis Pharmaceuticals, who will be conducting the screen in collaboration with several academic groups.

### Meeting reviews

Spring/Summer this year was a busy time for meetings. Accordingly, we have brought you a selection of brief reports from three of the most relevant meetings this season.

Karin van de Sande reports on the *Arabidopsis* Research meeting held in Madison in June, Andy Hayes reviews the Microarray Data Standards meeting which took place in Heidelberg this May and Mark Strivens covers the 'Genome Sequencing and Biology' meeting held at the Cold Spring Harbor Laboratories in May, at which the 'Gene-Sweepstake' on the number of human genes was initiated.

### Website review — UK CropNet

This website provides a comprehensive, UK-based resource and ideal first port of call for all those interested in crop plant genomics. The site provides access to several UK-based databases, including the *Arabidopsis* Genome Resource (AGR) and mirrors a wide range of databases hosted at the Cornell University site, such as RiceGenes. The site also provides a selection of comparative mapping tools, including tools that allow users to display whole genome comparisons as Oxford grids or real 'crop circles'.

Copyright © 2000 John Wiley & Sons, Ltd.

